# *TFG* mutation induces haploinsufficiency and drives axonal Charcot–Marie–Tooth disease by causing neurite degeneration

**DOI:** 10.1111/cns.13943

**Published:** 2022-08-19

**Authors:** Xihui Chen, Fangfang Liu, Kun Chen, Yufeng Wang, Anan Yin, Xiaowei Kang, Shanming Yang, Hanwen Zhao, Songqi Dong, Yunqing Li, Jing Chen, Yuanming Wu

**Affiliations:** ^1^ Department of Biochemistry and Molecular Biology, School of Basic Medicine Air Force Medical University Xi'an China; ^2^ Shaanxi Provincial Key Laboratory of Clinic Genetics Air Force Medical University Xi'an China; ^3^ Department of Neurobiology, School of Basic Medicine Air Force Medical University Xi'an China; ^4^ Department of Anatomy, Histology and Embryology and K.K. Leung Brain Research Centre, School of Basic Medicine Air Force Medical University Xi'an China; ^5^ Medical Genetics Yan'an University Yan'an China; ^6^ Department of Neurosurgery, Department of Plastic surgery, Xijing Institute of Clinical Neuroscience, Xijing Hospital Air Force Medical University Xi'an China; ^7^ Department of radiology Xi'an people's hospital (Xi'an fourth hospital) Xi'an China; ^8^ Student Brigade Air Force Medical University Xi'an China

**Keywords:** axonal Charcot–Marie–Tooth, haploinsufficiency, neurite degeneration, tropomyosin‐receptor kinase‐fused gene, zebrafish model

## Abstract

**Aims:**

*TFG*‐related axonal Charcot–Marie–Tooth (CMT) disease is a late‐onset, autosomal dominant, hereditary motor, and sensory neuropathy characterized by slowly progressive weakness and atrophy of the distal muscles. The objective of this study was to determine the common pathogenic mechanism of *TFG*‐related CMT type 2 (CMT2) caused by different mutations and establish a direct association between TFG haploinsufficiency and neurodegeneration.

**Methods:**

Three individuals carrying the TFG p.G269V mutation but with varying disease durations were studied. The effect of the p.G269V mutation was confirmed by analyzing protein samples extracted from the blood of two individuals. The functional consequences of both CMT2 mutant gene products were evaluated in vitro. The effect of TFG deficiency in the nervous system was examined using zebrafish models and cultured mouse neurons.

**Results:**

Overexpression of p.G269V TFG failed to enhance soluble TFG levels by generating insoluble TFG aggregates. TFG deficiency disrupted neurite outgrowth and induced neuronal apoptosis both in vivo and in vitro and further impaired locomotor capacity in zebrafish, which was consistent with the phenotype in patients. Wnt signaling was activated as a protective factor in response to TFG deficiency.

**Conclusion:**

CMT2‐related *TFG* mutation induces TFG haploinsufficiency within cells and drives disease by causing progressive neurite degeneration.

## Graphical abstract

*TFG* mutation in certain domain induces CMT2 by a TFG haploinsufficiency mechanism. The insufficient TFG expression then induces progressive neurite degeneration and causes CMT2‐liked motor disturbance in zebrafish models, which highlighted the critical role of TFG dosage in the maintenance of the neuronal system, and this process is companied by the activation of Wnt signaling.

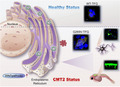

## 1 INTRODUCTION

Charcot–Marie–Tooth (CMT) disease is a clinically and genetically heterogeneous group of hereditary motor and sensory neuropathies characterized primarily by slowly progressing weakness, atrophy of the distal muscles, and depressed tendon reflexes.^1^ CMT is classified into two main subgroups based on nerve conduction velocity: CMT type 1 (CMT1, less than 38 m/s) with pathologic evidence of demyelination, and CMT type 2 (CMT2, over 38 m/s) with axonal damage.^2^

Charcot–Marie–Tooth is one of the most common hereditary neuromuscular disorders with a global prevalence of 1:2500; over 100 genes cause CMT through different patterns of inheritance.^1^, ^3^ The autosomal dominant forms are the most common, but autosomal recessive and X‐linked subtypes also occur.^4^ The recently identified tropomyosin‐receptor kinase fused gene (*TFG*) is related to the autosomal dominant CMT2 subtype.^5^

*TFG* is a fusion partner of the neurotrophic tyrosine kinase receptor for nerve growth factors and contributes to the generation of oncogenic products in multiple types of cancer.^6^ Recent studies revealed that TFG protein, localized to the endoplasmic reticulum (ER) exit site, modulates COP‐II‐dependent collagen secretion by interacting with SEC16 on the ER membrane.^7^ Structural analysis predicts three functional domains in the TFG protein. The Phox/Bem 1p (PB1) domain and coiled‐coil (CC) domain are responsible for facilitating protein oligomerization and driving oncogenesis^8^, ^9^; the proline and glutamate‐rich (P/Q rich) domain contributes to the proper formation of ER exit sites and intracellular protein homeostasis.^10^, ^11^

The p.P285L (Proline at site 285 is replaced by Leucine in TFG reference protein sequence) mutation, discovered in a patient with hereditary motor and sensory neuropathy with proximal dominant involvement (an atypical axonal CMT), established a link between *TFG* mutations and neurodegenerative diseases.^5^ p.R106C and p.G269V in TFG are associated with spastic paraplegia 57 (SPG57) and CMT2, respectively; these mutations cause neurological disorders through distinct pathogenic mechanisms. p.R106C is a loss‐of‐function mutation in the *TFG* gene that disrupts the ER architecture; the p.P285L mutation TFG causes ER stress by a gain of toxic function.^10^ In vitro studies examining the role of the TFG p.G269V mutant involved in CMT2‐mediated pathology reveal that the p.G269V mutant protein causes a dominant‐negative effect by trapping wild‐type TFG into cytoplasmic aggregates.^12^ These coaggregates were nontoxic to cells, but the level of wild‐type TFG was reduced, indicating that haploinsufficiency may be the underlying mechanism. Recently, a likely pathogenic *TFG* mutation, c.793C > G (p.P265A), was reported in a CMT2 family, and its pathogenic mechanism has never been studied.^12^

Length‐dependent axonal degeneration contributes to clinical impairment in CMT2 and other neurological disorders.^14^ However, the effect of TFG deficiency in the nervous system is unclear because of a lack of preclinical (animal and in vitro) models.

In the present study, we confirmed that p.G269V and a novel p.P265A mutation share the TFG haploinsufficiency mechanism that causes CMT2 in patients. Moreover, we used zebrafish models and mouse primary neuron cell cultures to establish a direct relationship between TFG haploinsufficiency and neurodegeneration.

## 2 MATERIALS AND METHODS

Written informed consents were obtained from all participants. The zebrafish model was established under technical and facility support of the Shanghai Model Organisms Center. All studies were approved by the ethics approval from the Air Force Medical University.

### 2.1 Subjects

Clinical evaluation of three individuals in the family was performed by two senior neurologists. Nerve conduction study and electromyography (EMG) were conducted on available individuals. Clinical evaluation was performed after the outbreak of COVID‐19. None of the participants in this study has been infected by Corona virus.

### 2.2 MRI studies

The hip and lower limbs of 2 individuals in the family (IV‐1, III‐2) were studied with MRI by using a 1.5‐tesla magnetic resonance unit. T1‐weighted images were used to represent muscle atrophy with fatty replacement.

### 2.3 Expression plasmids

A fragment containing the full‐length coding region of TFG was subcloned into the pcDNA3.1‐Myc and pcDNA3.1‐3xFlag vector to generate Flag‐tagged or Myc‐tagged TFG expression plasmids. The c.793C > G (p.P265A), c.806G > T (p.G269V), and c.854C > T (p.P285L) mutations were, respectively, introduced into the expression plasmids with Myc‐tag by using QuickMutation™ Site‐Directed Mutagenesis Kit (Beyotime, D0206).

### 2.4 Cell culture and transfection

The HEK293T cells were incubated in DMEM medium supplemented with 10% fetal bovine serum in a humidified incubator at 37°C under 5% CO_2_. Transfection of HEK293T cells with wild‐type or mutated‐TFG plasmids was performed using X‐tremeGENE HP DNA Transfection Reagent (Roche).

### 2.5 Zebrafish spinal motor neurons studies

The *hb9*:eGFP transgenic zebrafish lines with motor neuron‐specific *hb9* promoter and GFP reporter construct were used in this study.^15^ *tfg* translation‐blocking morpholino (ATG‐MO), splice‐blocking morpholino (E3I3‐MO), and standard control morpholino (control‐MO) were designed and produced by Gene Tools, LLC (http://www.gene‐tools.com/). MOs were injected into fertilized one‐cell stage embryos according to standard protocols.^16^ RT‐PCR was performed to confirm the efficacy of the E3I3‐MO. *ef1α* was used as a loading control. The MO sequences and primers sequences were listed in Tables S1 and S2. Embryos were dechorionated at 54 h postfertilization (hpf), anesthetized with 0.016% tricaine methanesulfonate (Sigma‐Aldrich) and then mounted on the lateral or dorsal side in a depression slide with 3% methylcellulose.

### 2.6 Zebrafish behavioral analysis

Ten larvae from each group were collected at 5‐dpf. Each larva was placed in a separate well of 96‐well plates and allowed to freely explore the aquarium for 30 minutes. The motion trail was tracked by a camera, and the digital tracks were analyzed by Ethovision XT software (Noldus Information Technology). Heatmap images were obtained using Nikon SMZ18 Fluorescence microscope and subsequently photographed with digital cameras. Movement indicators were calculated using image‐based morphometric analysis (NIS‐Elements D4.6, Japan).

### 2.7 Mouse primary neuron culturing and lentivirus transduction

Mouse primary neuron culturing was performed as described previously with some modifications.^17^ In brief, the cortical cortex from E14 mouse embryos was isolated and cut into small pieces. Blew the tissue pieces into suspension using a pipettor in the neurobasal medium supplemented with B27 and L‐Glutamine. Seed the single‐cell suspension into dishes precoated by Poly‐L‐Lysine (Sigma, P1399). rLV‐U6‐sh*Tfg*‐CMV‐mcherry‐WPRE and rLV‐U6‐shNC(scramble)‐CMV‐mcherry‐WPRE (Brainvta Company, China) were transduced into the cells after 24 hours. shRNA sequences were listed in Table S3. For the knockin experiments, rLV‐EF1α‐TFG (wild‐type)‐Myc‐P2A‐mcherry‐WPRE, rLV‐EF1α‐TFG (p.G269V)‐Myc‐P2A‐mcherry‐WPRE, or rLV‐EF1α‐TFG (p.P265A)‐Myc‐P2A‐mcherry‐WPRE were transduced into the cells after 24 hours' seed. The culture medium was changed after 12 hours of transduction. Half the amount of medium was changed every 2 days in the following cultivation. For inhibiting Wnt signaling pathway, 3 μM LGK974 was added to neurons on day 4 in culture. Change the culture medium after an hour's treatment and culture the neurons for another 4 days.

### 2.8 Statistical analysis

Data were analyzed using GraphPad Prism 8 software. All analyzed data exhibited a normal/Gaussian distribution using the Shapiro–Wilk test. Statistical significance was calculated using the Student's *t*‐test or ANOVA as appropriate. Data are presented as mean ± SD or SEM. Asterisks indicate the level of statistical significance, *p < 0.05, **p < 0.01, ***p < 0.001, ****p < 0.0001.

## 3 RESULTS

### 3.1 Clinical data of patients with different disease durations and genetic analysis

The proband was a 35‐year‐old female with a 10‐year history of weakness in her right lower limb. The left lower limb was affected 3 years after onset and symptoms gradually worsened with age. Over the last year, the patient developed weakness in her right hand. Here, electromyography data (Table S1) showed neurogenic lesions in the muscles innervating the medulla oblongata, cervical spine, thorax, and lumbar with reduced motor and sensory nerve conduction velocities. The patient lacked tibial reflexes on both sides. Her 12‐year‐old daughter, however, showed no neurological abnormalities. Blood chemistry data revealed no other abnormalities in addition to lightly decreased high‐density lipoprotein levels both in the proband and her daughter (Table S2). Family history of the proband showed that her 58‐year‐old uncle exhibited similar symptoms but with an age‐of‐onset in his thirties. The weakening of the lower limb has progressed to the point where the patient now requires a wheelchair for mobility. The primary diagnosis of this patient was peripheral neuropathy.

**FIGURE 1:**
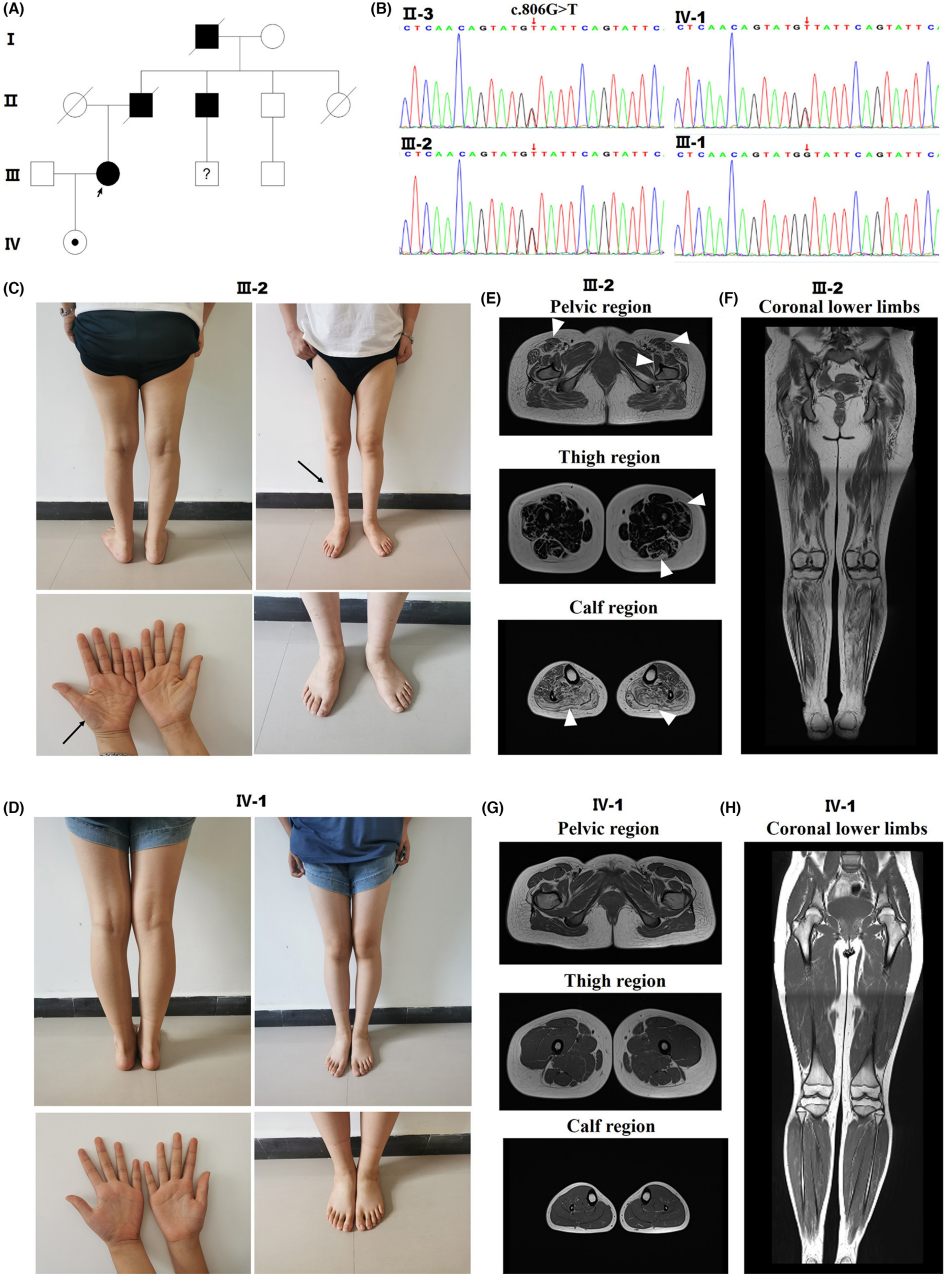
Detection of TFG p. G269V in a CMT2 family and clinical data of CMT patients with different disease durations. Pedigree of the CMT2 family. (B) DNA sequence chromatograms showing the heterozygous c.806G > T mutation in *TFG* present in II‐3, III‐2, and IV‐1 but not in III‐1. (C) Photographs of the proband (III‐2), a 35‐year‐old woman with a disease duration of 11 years. (D) Photographs of the proband's daughter (IV‐1), a 12‐year‐old girl whose disease has not yet appeared. (E) MRIs of III‐2 demonstrate widespread muscle atrophy and fat infiltration into the thighs (milder degree in the sartorius, rectus femoris, and tensor fascia lata and gluteus maximus, iliopsoas, and vastus lateralis; severe in gastrocnemius and soleus muscles) (arrowhead). (F) MRIs of IV‐1 depict normal muscle volume in the thighs. (G‐H) Coronal MRI images of lower limbs of III‐2 and IV‐1.

Exome sequencing was performed on the proband to obtain a definitive diagnosis. This analysis revealed a previously reported heterozygous missense CMT2 mutation c.806G > T (p.G269V) in the *TFG*. Sanger sequencing confirmed the c.806G > T allele in the proband, in her uncle, and in her asymptomatic daughter. The patient's husband did not carry the mutation (Figure 1A,B).

Table S3 summarizes the clinical physical examination results of the three family members carrying the CMT2 mutation with the following varying disease durations, respectively: not yet manifested, 10 years, and 28 years. The varying degrees of muscle weakness and muscle atrophy in the three individuals clearly indicated that the disease progresses with age. Photographs of the proband's limbs showed an apparent reduction in the volume of the thenar muscles and a smaller calf circumference (III‐2). The patient's daughter (IV‐1) showed a normal phenotype (Figure 1C,D). Magnetic resonance imaging (MRI) was then performed on the proband and her daughter. In the proband, symmetric fat replacement and muscle atrophy were found in the entire lower limb, including the pelvic, thigh, and calf regions; the phenotype was considerably more prominent in distal than in proximal muscles, with a moderate degree in sartorius, rectus femoris, and gluteus maximus muscles but most severe in the gastrocnemius and soleus muscles (Figure 1E,G). The proband's daughter, however, exhibited normal muscle volume in her lower limb (Figure 1F,H).

### 3.2 Spectrum of *TFG* mutations in humans and characterization of *Tfg* across species

**FIGURE 2:**
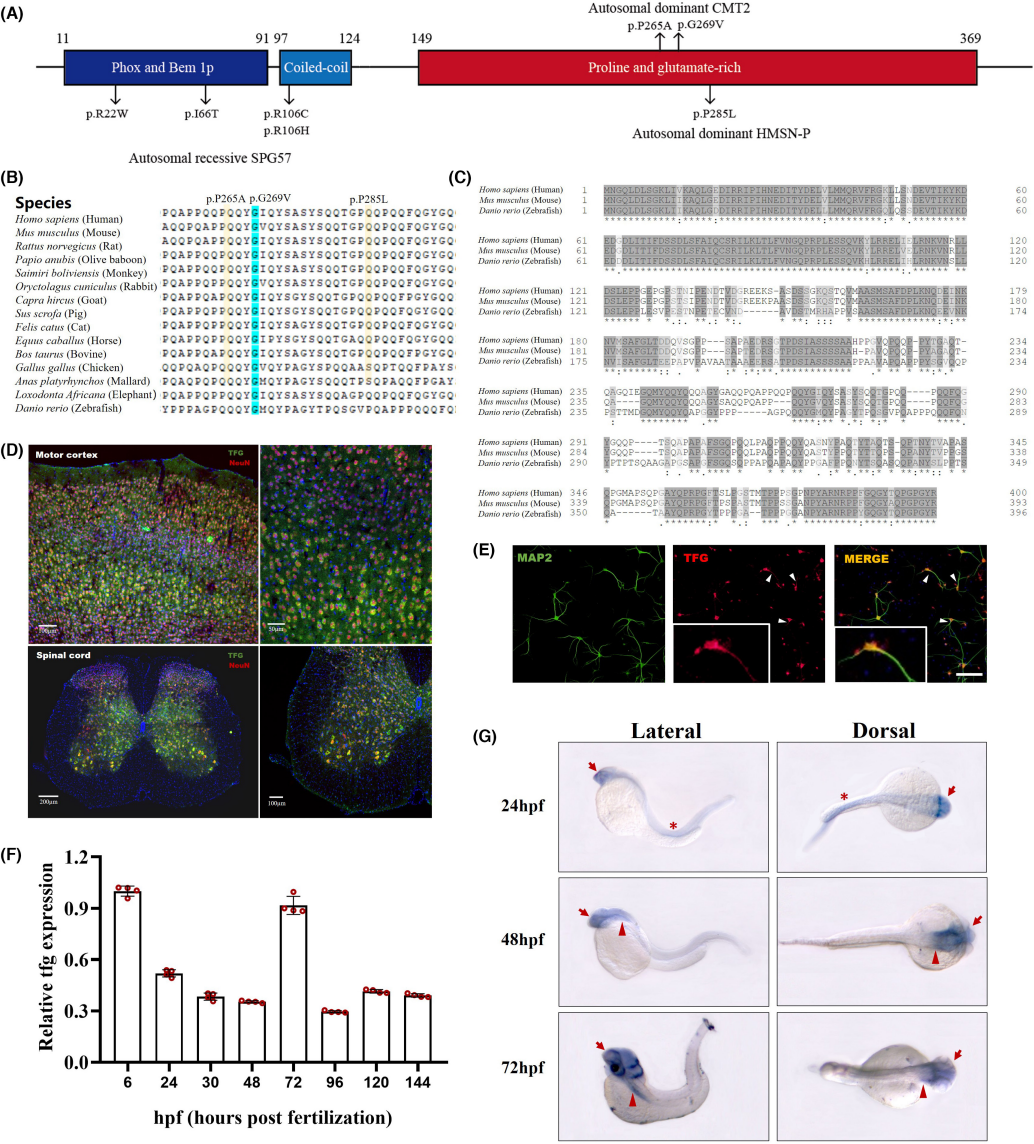
Spectrum of TFG mutations in human and characterization of *Tfg*. Linear map of the reported disease‐related mutations in TFG protein. (B) Conservation analysis of the substituted amino acid at 265,269 and 285 in TFG peptides. (C) Multiple‐sequence alignment of the TFG protein sequence in human, mouse, and zebrafish. (D) In situ hybridization histochemistry staining of coronal sections from adult mouse motor cortex and spinal cord. *Tfg* mRNA was labeled with specific probe (green) and neurons were labeled with NeuN antibody (red), nuclei were labeled with DAPI (blue). Scale bars: 200 μm, 100 μm, or 50 μm. (E) Immunofluorescence staining of primary cultured neurons at day‐8 in culture. The neurites were labeled with MAP2 (green) and the nucleus with DAPI (blue). White arrowhead shows TFG expression in the proximal dendrites of neurons. Scale bars: 50 μm. (F) qRT‐PCR for six embryo development stages (6, 24, 72, 96, 120 and 144 hpf) of wild‐type zebrafish embryos. Hpf, hours postfertilization. (G) Hybridization in situ of *tfg* in the whole zebrafish embryos at 24, 48 and 72 hpf. The arrow indicates the positive signal in the brain and eye region; the asterisk shows the positive signal in the spinal cord and notochord; the arrowhead shows the positive staining in the pectoral fin anlage.

Thus far, seven neurological disorder‐related mutations in *TFG* have been identified (Figure 2A). p.R22W and p.I66T, found on the PB1 domain, and p.R106C/H located on the CC domain were reported to cause autosomal recessive SPG57. p.P285L mutation in the P/Q‐rich domain causes autosomal dominant hereditary motor and sensory neuropathy with proximal dominancy (HMSN‐P), which is an atypical type of CMT. The p.G269V mutation discovered in this study was reported to occur in a CMT2 family.^12^ Furthermore, a possibly pathogenic p.P265A (c.793C > G) mutation was recently reported in patients with CMT2.^13^ These three CMT‐related mutations are localized to the P/Q‐rich domain, and the substituted amino acids are highly conserved throughout evolution (Figure 2B).

To further understand TFG characterization, we performed multiple‐sequence alignment of TFG protein among species. Our results showed that the key functional domains of the TFG protein are relatively conserved among human, mouse, and zebrafish (Figure 2C). A previous study has reported that TFG is expressed early in the mouse embryo with neural tissue‐specific staining, with a moderate level in the brain and a lower level in the primitive spinal cord.^18^ On this premise, we further examined TFG expression in the adult mouse nervous system. Our results showed that TFG is widely expressed in the cerebral cortex. Enlargement of the motor cortex and spinal cord anterior horn showed more TFG colocalization with NeuN than other regions, which indicated higher TFG expression level in motor‐related neurons (Figure 2D, Figure S1A). Immunofluorescent staining of TFG and the microtubule‐associated protein 2 (MAP2) in primarily cultured mouse neurons showed that TFG expresses mainly in the cell body and has a tiny amount of expression in the proximal dendrites of cortical neurons (Figure 2E). Similar to the expression pattern in mice, *Tfg* expression in zebrafish begins at a very early stage and continues throughout embryonic development, with substantially higher levels at 6 and 72 hpf (Figure 2F). In situ hybridization of entire zebrafish revealed that *tfg* was specifically expressed in the central nervous system (CNS) during development; this occurred mainly in the brain and eye, followed by the spinal cord and notochord, and at a tiny level in pectoral fin anlage from 48 hpf (Figure 2G). Immunofluorescence results show that TFG expresses specifically in the brain and spinal cord in both juvenile and adult zebrafish (Figure S1B).

### 3.3 CMT2‐related p.G269V *TFG* mutation induces reduced functional TFG expression level by forming insoluble cytosolic aggregates

**FIGURE 3:**
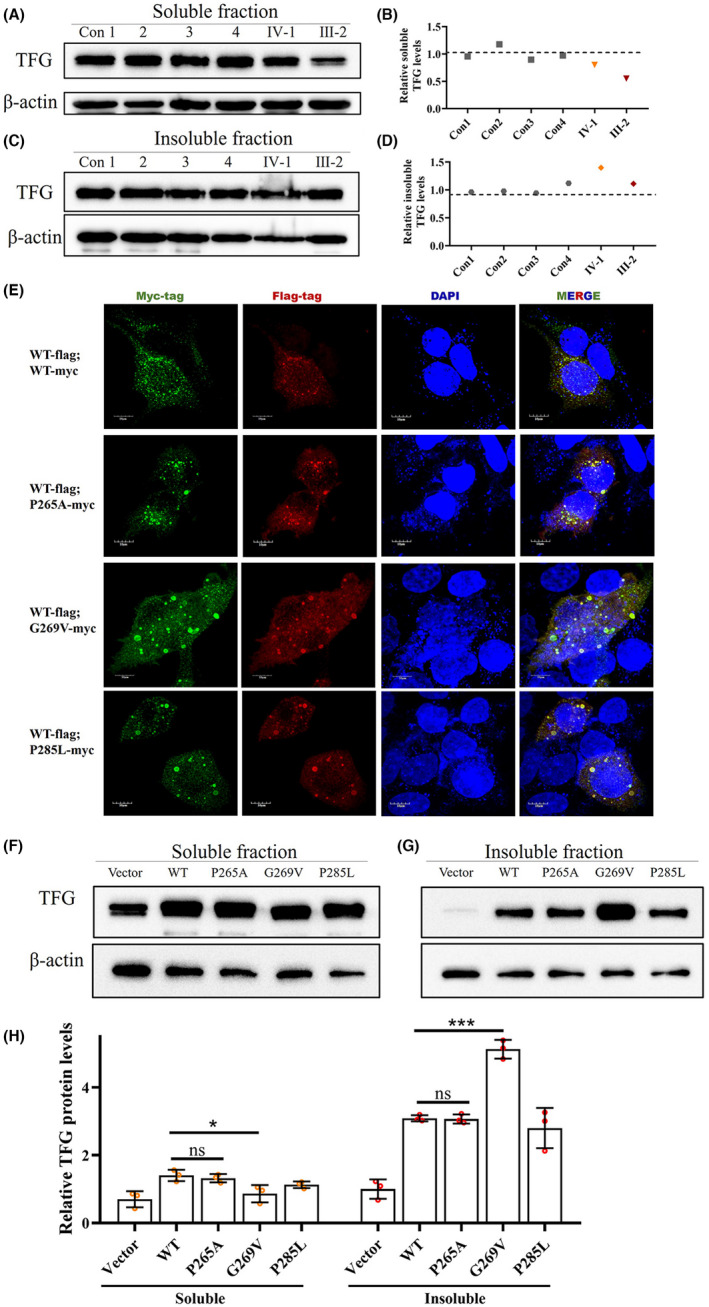
p.G269V TFG mutation induces reduced functional TFG expression level by forming insoluble cytosolic aggregates. (A and B) Western blot for soluble TFG extracted from PBMCs of four healthy controls and the two mutation carriers. Actin was used as the loading control. (C and D) Western blot for insoluble TFG extracted from PBMCs of four healthy controls and two mutation carriers. Actin was used as the loading control. (E) Immunofluorescence staining of HEK293T cells co‐transfected with plasmids encoding FLAG‐tagged wild‐type TFG (Red) and Myc‐tagged wild‐type TFG, Myc‐tagged p.P265A, Myc‐tagged p.G269V, and Myc‐tagged p.P285L TFG mutants (Green). Cell nuclei were labeled with DAPI (blue). Scale bar, 10 μm. (F) Representative western blot analysis of TFG expression in HEK293T cells transfected with plasmids expressing wild‐type TFG, p.P265A, p.G269V, and p.P285L TFG mutants; empty vector was used to represent endogenous TFG as a control. β‐actin was used as the loading control. (G) Quantification of F, mean ± SD; two‐tailed unpaired *t*‐test; ns, nonsignificant; **p  < 0.01; ***p < 0.001; ****p < 0.0001; *n* =  3.

A previous study suggested that p.G269V mutation can form nonfunctional and insoluble TFG aggregations in the cytoplasm.^12^ We tested this effect by measuring soluble and insoluble TFG protein levels in p.G269V carriers and healthy controls from the CMT2 family. Soluble TFG was slightly reduced in IV‐1 who carries the mutation but does not phenotypically express the disease (Figure 3A,B) and was further decreased in the proband who has had the condition for 11 years (Figure 3C,D).

To further confirm the aggregation hypothesis, we overexpressed the mutants in HEK293T cells. Co‐expression of Flag‐tagged wild‐type TFG and Myc‐tagged wild‐type TFG showed numerous colocalized punctate staining throughout the cytosol. The p.P285L mutant was used as a reference because the effect of this mutant has been fully studied both in cell lines and in patients' samples.^12^, ^19^, ^20^ In this study, cells co‐expressing Flag‐tagged wild‐type TFG and Myc‐tagged p.P285L TFG showed several bigger punctate, which is consistent with the previous studies.^12^, ^19^ However, Myc‐tagged p.G269V TFG co‐expressed with Flag‐tagged wild‐type TFG showed numerous larger cytoplasmic colocalized aggregates, which suggested that p.G269V mutant protein obtained greater self‐aggregation capacity, and could also aggregate with wild‐type TFG (Figure 3E). The p.P265A mutant did not show any larger aggregations. The phenotype was further confirmed in mouse primary cultured neurons (Figure S2).

We then evaluated by western blot whether these three TFG mutation alleles enhance the steady‐state soluble cellular TFG products. Overexpression of wild‐type TFG and p.P285L mutant both increased soluble TFG expression than empty vector expression in HEK293T cells, consistent with a previous study.^21^ Overexpression of the p.P265A mutant also increased soluble TFG levels similar to that of wild‐type TFG, which indicated that the mutation did not affect protein expression. p.G269V, however, did not show a full overexpression effect of soluble TFG level compared with the wild‐type but induced an increased TFG level of the insoluble fraction (Figure 3F,G).

Both our immunofluorescent staining and western blot results indicated that the p.G269V mutant allele could not produce a full copy of functional TFG protein. This means that a person who carries the p.G269V mutation in the genome would have a lower TFG protein expression level than wild‐type controls, an effect already confirmed in our study. Furthermore, an online program (https://www.deciphergenomics.org/) predicted that the *TFG* is more likely to be a dosage‐sensitive gene that may exhibit haploinsufficiency, a condition in which a single functioning copy of a gene is inadequate to maintain normal function.^22^

### 3.4 TFG deficiency causes neurite degeneration and increases neuronal apoptosis

To determine whether the mutants gained any toxic effect, we introduced the mutant TFG into primary cultured mouse neurons by transducing p.G269V TFG or p.P265A TFG lentivirus. Our results showed no changes in neurite length or apoptotic cell ratio in the p.G269V knockin neurons in comparison with the vector group and wild‐type knockin group, which indicated no cytotoxicity of the p.G269V TFG mutant. However, slightly reduced neurite length and an increased apoptotic cell ratio were observed in p.P265A knockin neurons, which suggested a gain of toxic function in this mutant (Figure S3). Taken together, the p.G269V and p.P265A may have different pathogenic mechanisms in inducing CMT2, and all of our current results have indicated that the p.G269V found in this study is a loss‐of‐function mutation that would lead to insufficient functional TFG levels in patients.

**FIGURE 4:**
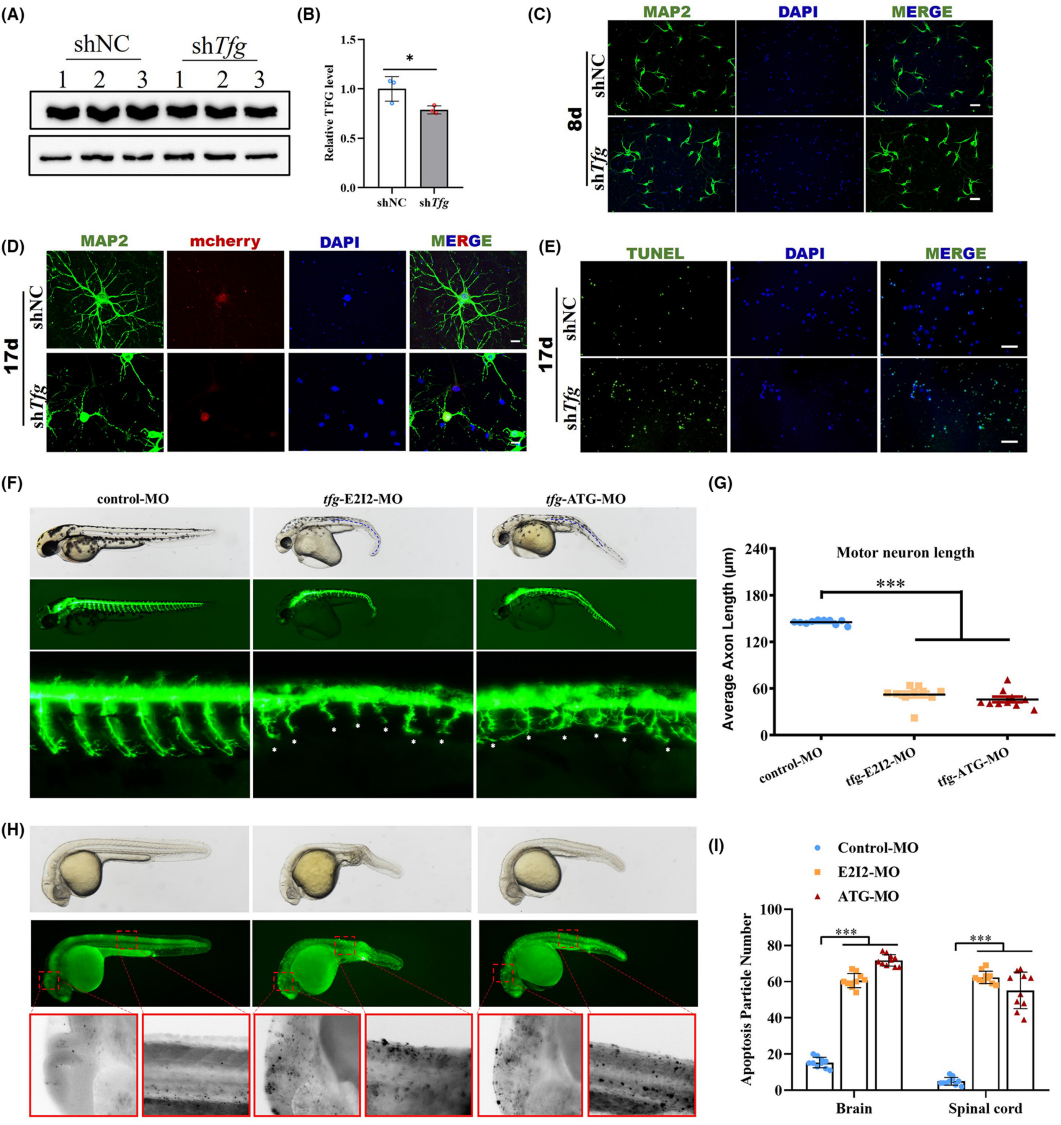
TFG deficiency impairs neurite outgrowth and causes neuronal apoptosis. (A and B) The efficiency of the small interfering RNA (siRNA) to suppress endogenous TFG expression was demonstrated by western blot. (mean ± SD; two‐tailed unpaired t‐test, *p  < 0.05; *n* =  3). (C) Immunofluorescence staining of primary cultured neurons on day 8 after *Tfg* knockdown. Neurites were labeled with MAP2 (green), and the nucleus with DAPI (blue). Scale bars: 50 μm. (D) Immunofluorescence staining of primary cultured neurons on day 17 after *Tfg* knockdown. Neurons were transduced with lentivirus with mCherry (red), neurites were labeled with MAP2 (green) and the nucleus was labeled with DAPI (blue). Scale bars: 20 μm. (E) TUNEL staining of control and *Tfg* knocked‐down neurons on day 17 in culture. (F) Gross morphology of zebrafish (*hb9*:eGFP) embryos at 2 dpf. Blue dotted lines show the curved body axis in both groups of *tfg* morphants. Dpf, days postfertilization. (G) Quantification of the average motor axon length. (mean ± SEM; ANOVA, ***p < 0.0001; *n* = 10). (H) Acridine orange staining of zebrafish at 32 hpf. Apoptotic cells are visible as bright green spots or black spots. The red dotted box shows the brain and spinal cord region of zebrafish. The boxed regions are shown at higher magnification on the bottom. (I) Quantification of apoptosis particle number in the brain and spinal cord (mean ± SEM; ANOVA, ***p < 0.0001; *n* = 10).

To investigate the effect of insufficient TFG levels in the nervous system, we sought to obtain neurons in which TFG expression was partially knocked‐down. We therefore transduced primary cultured mouse neurons with a mCherry‐sh*Tfg* lentivirus and examined the knockdown effect on day 8 after transduction (Figure 4A,B). An antibody against MAP2 was used to track neurite morphogenesis with immunofluorescence. On day 8 after *Tfg* knockdown, no obvious changes were observed in neurites between controls and the *tfg*‐knockdown group, suggesting that TFG deficiency did not affect neuronal maturation (Figure 4C). Neurite degeneration was observed on day 17 post‐transduction (Figure 4D). TUNEL assays revealed high levels of neuronal apoptosis on day 17 caused by TFG deficiency (Figure 4E).

We further established *tfg*‐knockdown zebrafish by microinjecting control and *tfg*‐targeted antisense morpholino oligonucleotides (MO) into fertilized one‐cell stage embryos (Figure S4). The motor neuron‐specific *hb9*:eGFP transgenic line was used to directly visualize spinal motor neurons. Zebrafish in the control group showed straight bodies and neatly arrayed motor neurons residing on the spinal cord at 2 days postfertilization (dpf). Both strains (I2E3‐MO or ATG‐MO) of the *tfg*‐knockdown zebrafish showed general malformations with a curved body axis and nonuniformly arranged motor neurons (Figure 4F). The lengths of spinal motor axons in *tfg*‐knockdown zebrafish larvae were significantly shorter than in controls (Figure 4G). Acridine orange staining results revealed high levels of neuronal apoptosis both in the brain and spinal cord of *tfg*‐knockdown zebrafish (Figure 4H,I).

The consistent results in mouse primary cultured neurons and zebrafish models indicate that TFG deficiency can cause neurite degeneration and increases neuronal apoptosis.

### 3.5 TFG deficiency reduces motor capacity and causes high mortality in zebrafish

**FIGURE 5:**
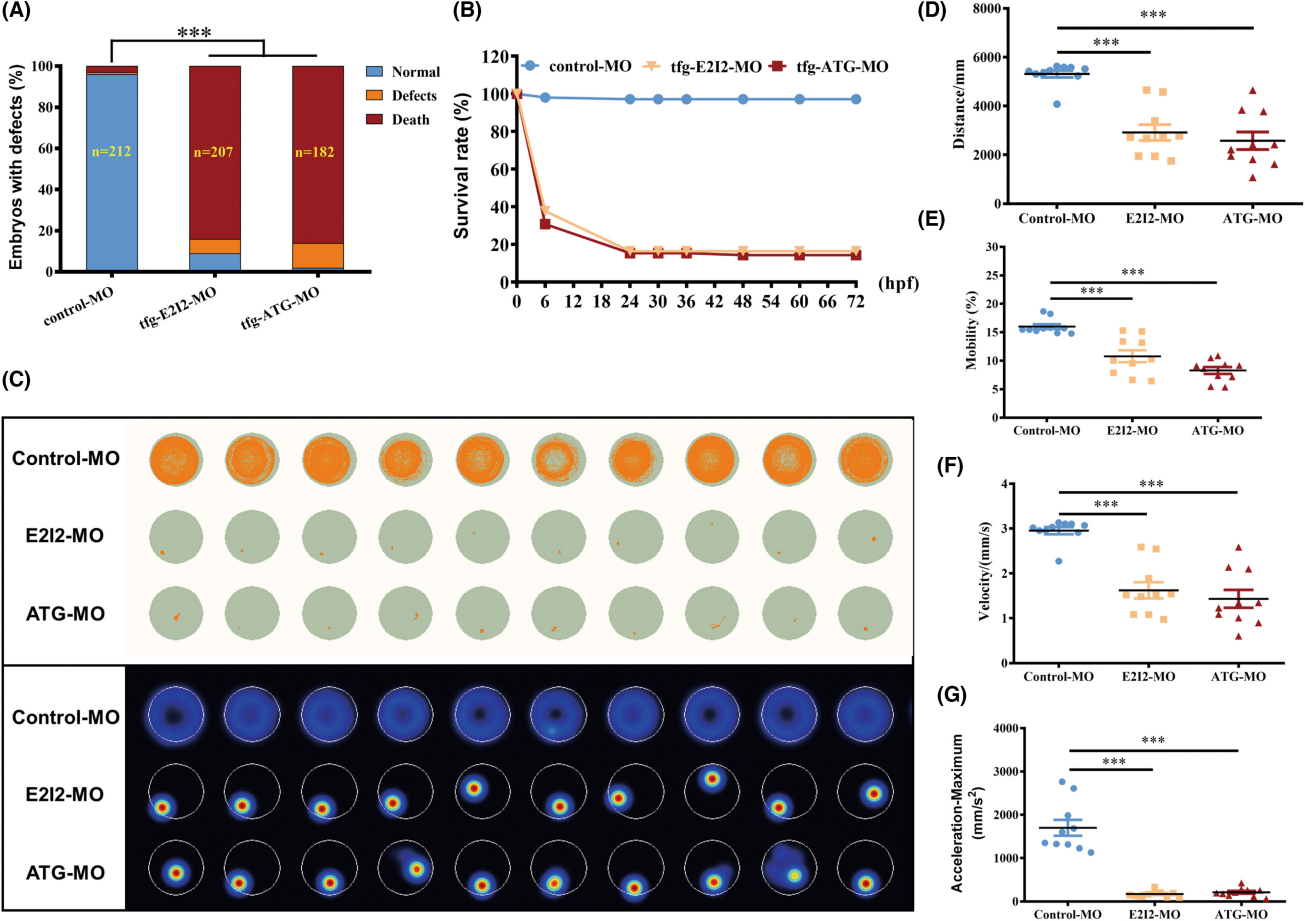
Locomotor capacity is reduced in *tfg* morphants. (A) Percentages of embryos with developmental defects in control versus *tfg* morphants at 3 dpf. (B) A time‐course plot of percent survival in control versus *tfg* morphants for 3 days. (C) Digital tracks and heatmap image in larvae from control‐MO‐ and *tfg*‐MO‐injected groups at 5 dpf. (D–G) Statistical analyses of the average total movement distance, mobility, velocity, and maximum acceleration in the *tf*g‐MO and control groups (mean ± SEM; ANOVA; ***p < 0.0001; *n* = 10). dpf, days postfertilization.

Within 3 days of MO injection, zebrafish in the control group showed a nearly 100% survival rate and normal development. Over 80% of zebrafish injected with *tfg*‐MO died within 3 days; those that survived had a high rate of deformity (Figure 5A). We calculated the survival rate of *tfg*‐deficient zebrafish at different time points within 72 hpf. The highest mortality occurred at 6 hpf when *tfg* expression was highest during development; the mortality rate decreased afterward and development stabilized at 24 hpf, leaving fewer than 20% of surviving zebrafish (Figure 5B).

Locomotor behavioral tests were then performed on 5 dpf control larvae and surviving *tfg*‐knockdown larvae. Digital tracks and corresponding heatmaps (Figure 5C) showed the decreased motor capacity of the *tfg*‐knockdown fish with a smaller total movement distance, lower velocity, reduced mobility, and smaller maximum acceleration than in control‐MO‐injected groups (Figure 5D–G). These data suggested that sufficient *tfg* expression contributes to motor neuron development and is required for normal locomotor function in zebrafish.

### 3.6 Wnt signaling is activated in response to TFG deficiency as a protective factor

Wnts are morphogenic molecules that act as instructive signals for axonal extension and neuroprotection during the neurodevelopmental process in vertebrates.^23^, ^24^ In models of Alzheimer's disease (AD), activation of Wnt signaling protects neurons against Aβ oligomer‐induced neurotoxic injuries.^25^, ^26^

**FIGURE 6:**
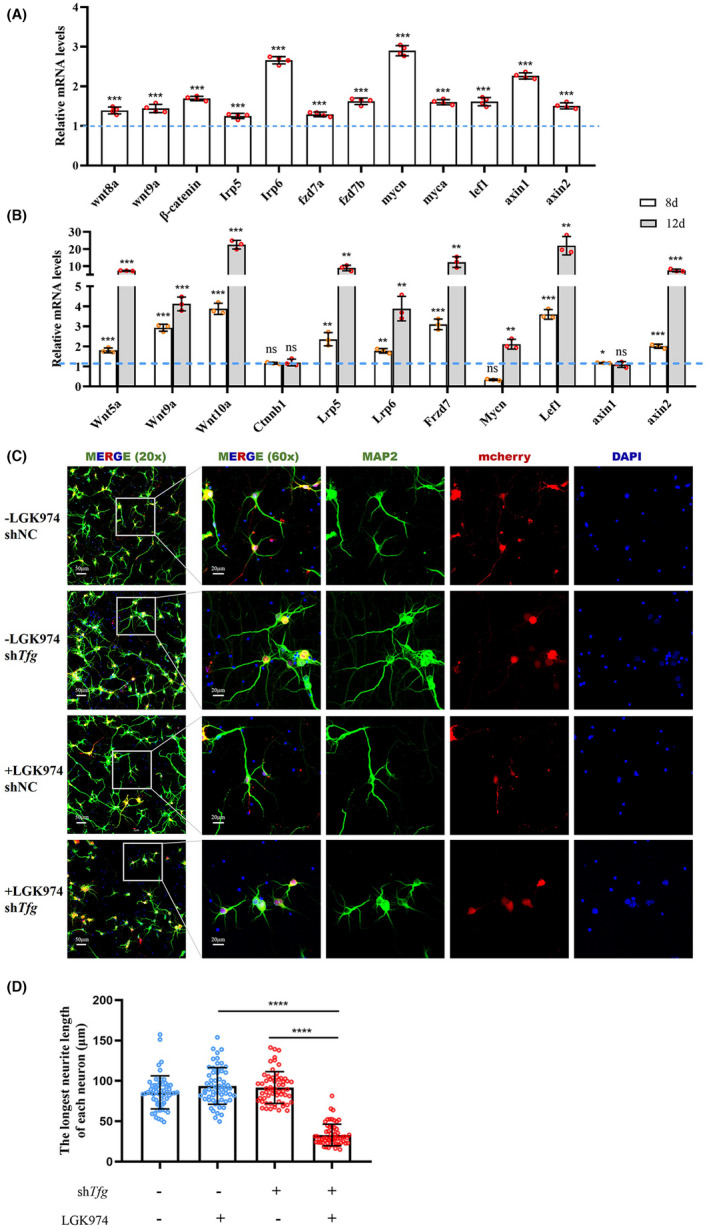
Wnt signaling is activated in response to TFG deficiency. (A) qRT‐PCR‐based validation of the key molecule in Wnt signaling in *tfg* morphants and control morphants at 3 dpf. (mean ± SD; two‐tailed unpaired *t*‐test, ***p < 0.001; *n* =  3). (B) qRT‐PCR‐based validation of the key molecule in Wnt signaling in primary cultured neurons on day 8 and 12 in culture after *Tfg* knockdown. (mean ± SD; two‐tailed unpaired *t*‐test; ns, nonsignificant; **p < 0.01; ***p < 0.001; *n* = 3). (C) Immunofluorescence staining of primary cultured neurons on day 8 after *Tfg* knockdown and pretreated with LGK974 for 24 h on day 4. Neurites were labeled with MAP2 (green) and the nucleus with DAPI (blue). Scale bars: 20 μm. (D) Quantification of the longest neurite length on day 8 in culture (mean ± SD; two‐tailed unpaired t‐test; ns, nonsignificant; ****p < .0001; *n* = 60).

To determine whether TFG deficiency promoted Wnt signaling, qPCR was performed on *tfg*‐knockdown zebrafish and mouse primary cultured neurons. In zebrafish at 5 dpf, *wnt8a* and *wnt9a* and the canonical co‐regulator of Wnt signal, β‐catenin, were upregulated in the *tfg*‐knockdown group. The Wnt ligand receptors *frzd* (*frzd7a* and *frzd7b*), and the coreceptor *lrp5/6*, were significantly upregulated in response to *tfg* deficiency. Expression of the downstream transcription factor *lef* and *wnt* target genes including *axin1*, *axin2*, *myca*, and *mycn* were also upregulated, indicating focal activation of Wnt signaling in *tfg*‐knockdown zebrafish (Figure 6A). We then investigated Wnt signaling in mouse primary cultured neurons following *Tfg* knockdown, at 8 and 12 days, respectively. *Wnt5a*, *Wnt9a*, and *Wnt10a* were upregulated in the *Tfg*‐knockdown group 8 days after transduction and were further elevated at 12 days. Expression of the receptors *Lrp5/6* and *Frzd7*, and downstream *Mycn*, *Lef1*, and *axin2* also increased according to this pattern (Figure 6B). We also tested these Wnt‐related molecules in knockin neurons. No change was found in either of the knockin models, which reconfirmed that Wnt signaling increased specifically in response to *Tfg* reduction but not to the mutant itself (Figure S5).

To further determine whether the increased Wnt signaling is neuroprotective in response to *Tfg* deficiency, we used LGK974, a WNT pathway inhibitor,^27^ to examine its effect on *Tfg‐*knockdown neurons. Immunofluorescent staining with MAP2 revealed that LGK974 significantly shortened the neurites length of *Tfg‐*knockdown neurons on day 8 after infection, while LGK974 itself did not affect the neurite outgrowth in control neurons (Figure 6C,D).

Taken together, the Wnt signaling pathway was activated by *Tfg*‐knockdown in direct proportion to impaired axonal outgrowth severity. It may act as a protective factor that facilitates neurite outgrowth under conditions of TFG deficiency.

## 4 DISCUSSION

*TFG* mutations associated with neurological disorders are classified into two categories based on inheritance patterns: autosomal recessive and autosomal dominant. All autosomal recessive mutations occur on the Phox and Bem1p and CC domain, and cause early‐onset SPG; autosomal dominant mutations localize to the PQ‐rich domain and cause middle‐aged onset axonal CMT or HMSN‐P.^27^ The distinct clinical phenotype in *TFG*‐related neurological disorders indicate a different pathogenic mechanism for various TFG mutations.

In the present study, the clinical and experimental results of p.G269V carriers suggested a progressive reduction in soluble TFG protein levels that correlated with disease progression. Our in vitro study further showed that the CMT2‐related p.G269V *TFG* mutant alleles could not produce a full copy of functional TFG protein because of forming insoluble TFG aggregates, thus arises a potential haploinsufficiency pathogenic mechanism in the patient carrying the mutation. However, the newly reported p.P265A mutation appears to play its pathogenic role through gaining toxic function, which will be further studied.

On the basis of our results for p.G269V, the TFG haploinsufficiency hypothesis was formed. Partially knockdown of the target gene is a common method to mimic the effect of haploinsufficiency, which was also the strategy we used in this study.^28^, ^29^

Zebrafish have a series of high conservation genes associated with neurodegenerative diseases in humans.^30^ For *tfg*, the specific CNS‐associated expression pattern in the zebrafish embryo indicates that zebrafish is a robust experimental model to study the functional significance of *tfg*. We hypothesized that haploinsufficiency contributes to CMT2 disease pathophysiology and proceeded to knock down *Tfg* in zebrafish and mouse neurons in primary culture. Substantial motor neuron degeneration and a reduction in motor ability in *tfg*‐knockdown zebrafish clarified the CMT2 phenotype observed in patients. In vitro, TFG deficiency did not affect neuron maturation but failed to maintain neurite growth and induced neurite degeneration at a later stage. These data may provide evidence as to why *TFG*‐related CMT2 patients have a common late‐onset and progressively deteriorating phenotype. Moreover, the knockin experiments indicate a gain of toxic function for the p.P265A mutant, suggesting that each of the three CMT‐related TFG mutations has a distinct pathogenic mechanism. In Table S4, we provide a summary of the phenotypes of these mutants, which would make the distinctions clear.

The late onset in both patients and the primary cultured neurons suggest that an endogenous protection mechanism against TFG reduction may exist. Our data show that the Wnt signaling pathway and its downstream molecules, including *Axin2*, are strongly activated following *Tfg*‐knockdown. LGK974 is reported to potently reduces Wnt‐dependent *Axin2* mRNA levels.^27^ In our study, the inhibitory effect on neurite outgrowth induced by LGK974 in *Tfg*‐knockdown neurons revealed Wnt‐dependent *Axin2* as a potential downstream molecule. Other studies have also reported that the Wnt signaling is involved in the maintenance of neuronal health in the adult brain and neuroprotection against neurodegenerative diseases such as AD and Parkinson's disease,^24^, ^31^, ^32^ and may, therefore, offer opportunities for novel therapies for *TFG*‐related CMT2.

## 5 CONCLUSION

In summary, we found a potential TFG haploinsufficiency mechanism for a CMT2‐associated *TFG* mutation and studied its effect by establishing both in vitro neuron models and in vivo zebrafish models. Progressive neurite degeneration and a CMT2‐liked motor disturbance were found in these TFG‐deficient models, which highlights the critical role of TFG dosage in the maintenance of the neuronal system.

## AUTHOR CONTRIBUTIONS

Y.W., Y.L., and J.C. supervised the project. X.C., F.L., and K.C. established the animal models and were major contributors in writing the manuscript. Western blotting and cell experiments were performed by Y.W., A.Y., and X.K. contributed to the acquisition of clinical data. S.Y. contributed to the staining experiments in zebrafish. H.Z. and S.D. performed immunofluorescence experiments. All authors read and approved the final manuscript.

## FUNDING INFORMATION

This work was supported by the National Natural Science Foundation of China (82071373 and 31570906); Key Research and Development Program of Shaanxi Province 2021SF‐088, 2020SF‐204, and 2019SF‐059; Key Innovative Project in Shaanxi, Grant/Award Number: 2021ZDLSF02‐02; Innovation Capability Support Program of Shaanxi, Grant/Award Number: 2021TD‐57.

## CONFLICT OF INTEREST

The authors declare that they have no competing interests.

## INFORMED CONSENT

Written informed consents were obtained from all participants.

## Supporting information


**Appendix S1** Supplementary Materials

## Data Availability

The datasets used and analyzed during the current study are presented in the figures, and they are available from the corresponding authors upon reasonable request.
