# Supplementary material for: TFG mutation induces haploinsufficiency and drives axonal Charcot–Marie–Tooth disease by causing neurite degeneration
Source: CNS Neurosci Ther. 2022 Aug 19;28(12):2076–89. doi: 10.1111/cns.13943 (PMC9627391; doi:10.1111/cns.13943)
Supplement: Supplementary file 1 — Appendix S1 Supplementary Materials [file CNS-28-2076-s001.pdf]

# Supplementary Materials

## Supplementary Methods

### Whole exon sequencing analysis and Sanger sequencing

Whole exon sequencing was performed on the proband. The qualified genomic DNA sample extracted from peripheral blood. Extracted DNA was then amplified by ligation-mediated PCR (LM-PCR), purified and hybridized to the NimblegenSeqCap EZ Library. IDT The xGen Exome Research Panel v1.0 was used for the enrichment of exonic and adjacent intronic sequences. The enriched samples were sequenced on the Illumina NovaSeq 6000 platform (Illumina, Inc., San Diego, CA). The sequenced reads were aligned to the human reference genome (GRCh38) using SOAP aligner. Detected variations that have been reported to be related to neuromuscular disease were verified in other members of the family by Sanger sequencing using an AB 3730xl DNA analyzer (Applied Biosystems). Sequencing results were analyzed with Chromas software (Technelysium Pty Ltd).

### Conservative analysis

The conservative analysis of single amino acid site was achieved by MEGA7 software. The TFG protein sequence comparison and the conserved domain architecture among human, mouse and zebrafish was performed using the online tool Uniprot (https://www.uniprot.org/).

### In situ hybridization histochemistry

Wile-type adult mice were deeply anesthetized with isoflurane and transcardially perfused with saline solution followed by 4% PFA. The harvested brains and spinal cord were postfixed in 4% PFA and dehydrated in 30% sucrose on the next day. 30 μm-thickness coronal sections were made using a cryotome. A complementary DNA fragment of Mus musculus Trk-fused gene transcript variant 1 (*Tfg*, NM_019678.3: 696-1448) was used as templates for the synthesis of digoxygenin labeled antisense RNA probes (DIG RNA Labeling Mix, Roche 11277073910 and MAXIscript T7/SP6 Transcription Kit, Invitrogen AM1322). In brief, mouse brain and spinal cord sections underwent standard pretreatment as previously described[1]. 2 ng/μl probes followed by Anti-Digoxigenin-POD were used for in situ hybridization histochemistry. Alexa Fluor 488-conjugated avidin (Life Technologies, A21370, 1:200) was used to lable *Tfg*. Samples then proceeded to regular

immunofluorescence using Anti- NeuN antibody (Cell Signaling Technology, 24307, 1:100) and Alexa Fluor® 594-conjugated AffiniPure Goat Anti-Rabbit IgG (H+L) (111-585-003). Zebrafish embryos were fixed with 4% PFA and dehydrated in gradient ethyl alcohol. A complementary DNA fragment of Danio rerio trafficking from ER to Golgi regulator transcript variant 2 (*tfg*, NM_001328573.1: 24-872) was used as templates for the synthesis of digoxygenin labeled antisense RNA probes. In situ hybridization on whole mount zebrafish embryos were carried out according to the online procedures (http://zfin.org/ZFIN/Methods/ThisseProtocol.html). Next day, the zebrafish embryos were incubated with alkaline phosphatase-conjugated anti-digoxigenin antibody (Roche 11093274910, 1:2500). Biotinylated tyramine-glucose oxidase amplification was carried out to amplify the signal for *tfg*. The zebrafish embryos were visualized with NBT/BCIP Stock Solution (Roche 11681451001, 1:50).

**Immunofluorescence**

Zebrafish were postfixed in 4% PFA and dehydrated in gradient ethyl alcohol. 4 μm-thickness sagittal sections were made using a microtome for immunofluorescence. Primary cultured neurons or cell lines were seeded on round glass coverslip in 24-well plates at a density of 2 x $10^5$ per well. Samples were fixed and proceed to regular immunofluorescence at indicated days after infection or 48 h transfection. The following antibodies were used: Anti-TFG (Abcam, ab156866, 1:250), Anti-MAP2 (Abcam, ab11267, 1:1000), Anti-Myc tag (Cell Signaling Technology, 9B11, 1:1000), Anti-Flag tag (Proteintech, 20543-1-AP, 1:100). Secondary Antibodies was purchased from Jackson ImmunoResearch: Alexa Fluor® 488-conjugated AffiniPure Goat Anti-Mouse IgG (H+L) (115-545-003). Alexa Fluor® 594-conjugated AffiniPure Goat Anti-Rabbit IgG (H+L) (111-585-003). Alexa Fluor® 488 AffiniPure Goat Anti-Rabbit IgG (H+L) (111-545-003).

**TUNEL assay**

Apoptotic neurons were detected by using the Fluorescein (FITC) TUNEL Cell Apoptosis Detection Kit (Servicebio, G1501). According to the instruction, cells seeded on the coverslips were fixed in 4% paraformaldehyde, rinsed with PBS, and then permeabilized by 0.1% Triton X-100. The reaction mixture was loaded to label the fragmented DNA of the

apoptotic cells by FITC. DAPI (Invitrogen, D1306) was used to label the cell nuclei. FITC-positive signals were detected under a fluorescence microscope.

**Acridine orange staining**

Control-MO injected embryos and embryos injected with *tfg* MO were immersed in 5 μg/ml AO (acridinium chloride hemi-[zinc chloride], Sigma-Aldrich) in fish holding-water for 60 min at 32-hpf. Next, zebrafish were rinsed thoroughly in fish holding-water three times (5 min/wash) and anaesthetized with 0.016% MS-222 (tricaine methanesulfonate, Sigma-Aldrich, St. Louis, MO). Zebrafish were then oriented on their lateral side and mounted with methylcellulose in a depression slide for observation by fluorescence microscopy. The apoptosis particle number at spinal cord was quantitatively analyzed.

**Western blot**

Primary cultured neurons were lysed with the RIPA buffer (Beyotime, P0013B). Peripheral blood mononuclear cells (PBMCs) from patients or healthy controls were isolated from heparinized blood by density-gradient centrifugation. HEK293T cells were collected after 48 h transfection. For soluble and insoluble protein extraction, collected cells were lysed with the RIPA buffer (Beyotime, P0013D) and centrifuged to generate the soluble fraction in the supernatant. The sediment was further solubilized using urea buffer (8 M urea, 50 mM Tris, 0.5 mM EDTA, 50 mM NaCl) to recover the insoluble fraction. The following primary antibodies were used: TRK fused gene antibody (Abcam, ab156866, 1:10000); Anti-Myc tag (Cell Signaling Technology, 9B11, 1:1000), Anti-Flag tag (Proteintech, 20543-1-AP, 1:2000); β-actin antibody (Sigma, A1978, 1:3000). All secondary antibodies were purchased from ZhuangzhiBio (anti-mouse, 1: 8000; anti-rabbit, 1:8000). Western blot quantification was performed using the ImageJ software.

**Quantitative real-time PCR**

Total RNA of zebrafish or primary cultured neurons were isolated from tissue or cultured cells using Multisource Total RNA Miniprep Kit (Axygen, 365) and then used for cDNA synthesis (PrimeScript™RT Master Mix, Takara, RR036Q) and amplification by real-time PCR according to the manufacturer's instructions (SYBR Premix Ex Taq™ II, Takara, RR820A). Relative gene expression quantification was based on the comparative threshold

cycle method ($2^{-\Delta\Delta Ct}$) using β-actin as an endogenous control gene. The primer information is provided in Supplementary Method table 2.

## References


1. Guo B, Chen J, Chen Q, Ren K, Feng D, Mao H, Yao H, Yang J, Liu H, Liu Y, Jia F, Qi C, Lynn-Jones T, Hu H, Fu Z, Feng G, Wang W, Wu S. Anterior cingulate cortex dysfunction underlies social deficits in Shank3 mutant  mice. Nat Neurosci 2019;22:1223-1234

## Supplementary Tables

**Supplementary Table 1 Muscle electrophysiologic data of individuals in the pedigree**

| Electromyography | IV-1 | III-2 |
|---|---|---|
| MNCV | | |
| Median.L | normal | normal |
| Median.R | normal | normal |
| Ulnar.L | normal | mildly reduced |
| Ulnar.R | normal | mildly reduced |
| Peroneal.L | normal | reduced |
| Peroneal.R | normal | reduced |
| Tibial.L | normal | reduced |
| Tibial.R | normal | reduced |
| Femoral.L | normal | reduced |
| Femoral.R | normal | reduced |
| SNCV | | |
| Median.L | normal | mildly reduced |
| Median.R | normal | mildly reduced |
| Ulnar.L | normal | mildly reduced |
| Ulnar.R | normal | mildly reduced |
| Superficial peroneal.L | normal | absent |
| Superficial peroneal.R | normal | absent |
| Sural.L | normal | absent |
| Sural.R | normal | absent |
| H - reflection | | |
| Tibial.L | normal | absent |
| Tibial.R | normal | absent |
| F-wave | | |
| Tibial.L | normal | low frequency |
| Tibial.R | normal | low frequency |

Abbreviations: .L= left; .R=right; MNCV = motor nerve conduction velocity; SNCV = sensory nerve conduction velocity;

**Supplementary Table 2 Blood chemistry data of individuals in the pedigree**

| Blood chemistry data | Ⅳ-1 | Ⅲ-2 |
|---|---|---|
| CK (normal: 25-173U/L) | 107 | 164 |
| FBS (normal: 3.89-6.11mmol/L) | 4.62 | 4.86 |
| Cholesterol (normal: <5.2mmol/L) | 3.63 | 3.93 |
| LDL (normal: 0-4.11mmol/L) | 2.15 | 2.27 |
| HDL (normal: >1.15mmol/L) | 1 | 1.09 |
| Triglyceride (normal: | 0.93 | 0.96 |

Abbreviations: CK, creatine phosphokinase; FBS, fasting blood sugar; LDL, low-density lipoprotein; HDL, high-density lipoprotein.

**Supplementary Table 3 Clinical manifestations of individuals in the pedigree**

| Characteristic | IV-1 | III-2 | II-3 |
|---|---|---|---|
| Gender | F | F | M |
| Age at study，years | 12 | 35 | 58 |
| Age at onset，years | - | 25 | 30+ |
| Muscle weakness | | | |
| Distal upper limbs | normal | left V-; right V- | mild |
| Proximal upper limbs | normal | normal | normal |
| Distal lower limbs | normal | left IV+; right V- | marked |
| Proximal lower limbs | normal | left IV; right IV- | marked |
| Muscle atrophy | | | |
| Distal upper limbs | normal | bilateral first dorsal interosseous and abductor digiti minimi | present |
| Proximal upper limbs | normal | normal | normal |
| Distal lower limbs | normal | bilateral tibialis anterior muscle | present |
| Proximal lower limbs | normal | normal | present |
| Sensation loss | no | no | no |
| Shrug | normal | normal | - |
| Neck rotation | normal | normal | - |
| Fasciculation | no | yes | no |
| Cramping | no | no | no |
| Hand tremor | no | yes | yes |
| Plantar response | normal | absent | - |
| Radioperiosteal reflex | + | normal(++) | - |
| Knee/anklle deep tendon reflex | normal(++) | left +; right absent | - |
| Pes cavus | no | suspicious | - |
| Gait (Ambulatory state) | normal | slow walking | wheelchair bound |

-Data not available

**Supplementary Table 4 Summary of the phenotype of wild-type TFG and its three CMT-related mutants**

| Genotype / Phenotype | Wild-type | p.P265A | p.G269V | p.P285L |
|---|---|---|---|---|
| Forming cytosolic aggregates | No | No | Yes | Mild |
| Increased insoluble TFG | No | No | Yes | No |
| cytotoxicity | No | Yes | No | Yes (Reported) |

## Supplementary Figures

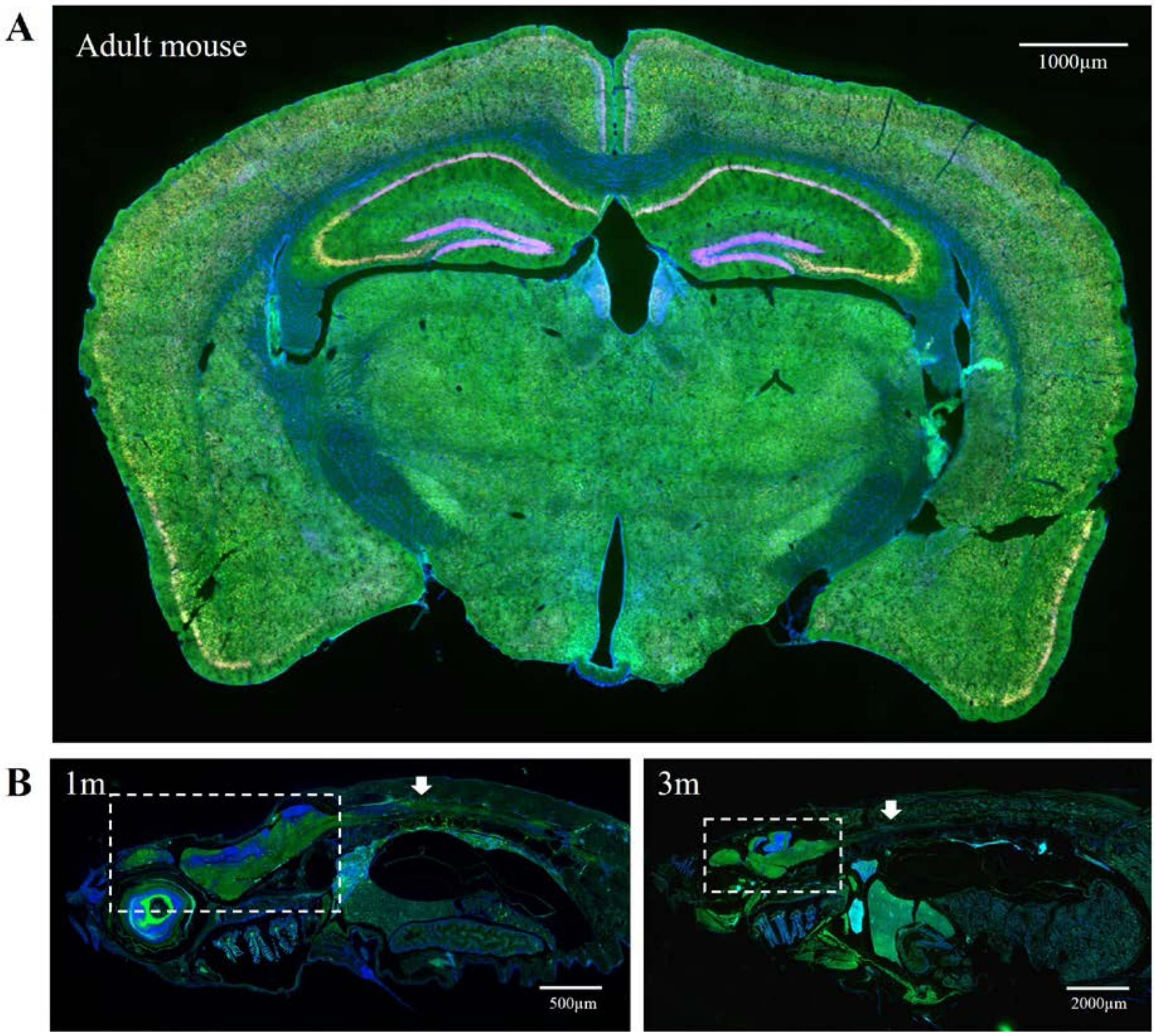


**Supplementary Figure 1. TFG expression pattern in mouse and zebrafish.**

(A) In situ hybridization histochemistry staining of coronal sections from adult mouse. *Tfg* mRNA was labeled with specific panel (green) and neurons were labeled with a NeuN antibody (red), nucleus were labeled with DAPI (blue). Scale bars: 200 μm, 100 μm or 50 μm. (B) Immunofluorescence staining of zebrafish sagittal sections at 1-month and 3-month-age. TFG protein was labeled with TFG antibody (green) and nucleus with DAPI (blue). White dashed boxes indicates the brain, white arrows indicate the spinal cord. Scale bars: 2000 μm or 500 μm.

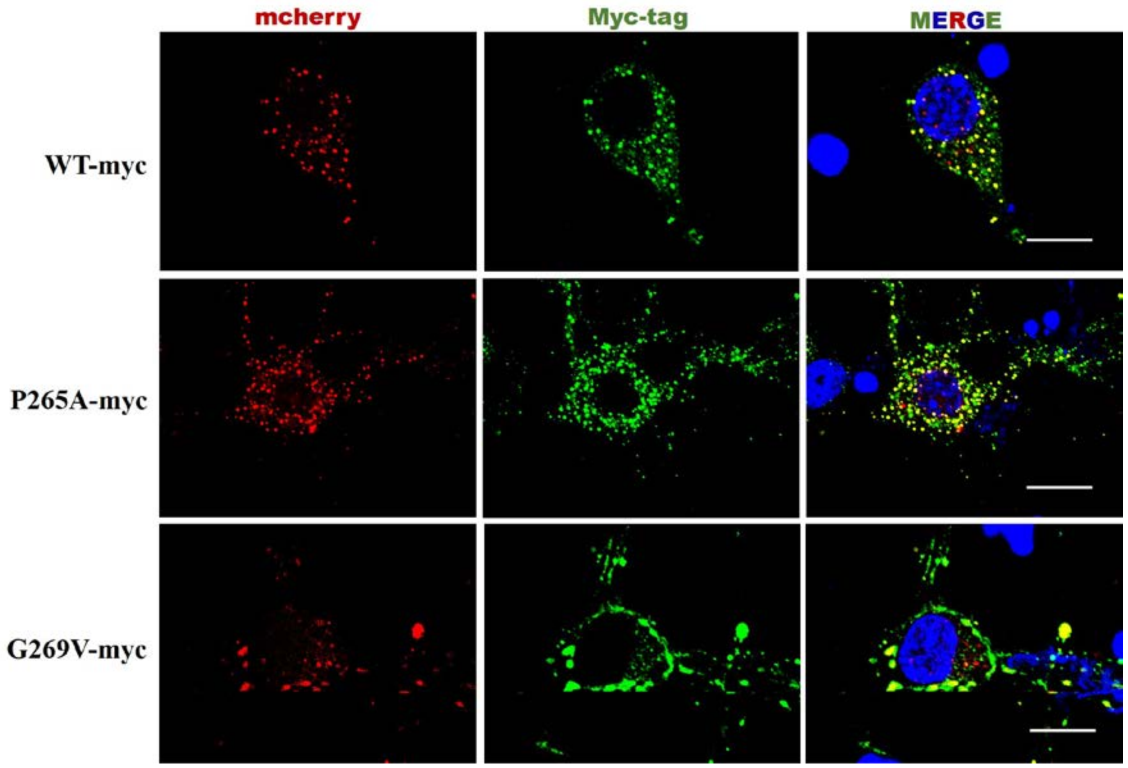


**Supplementary Figure 2. p.G269V *TFG* mutation induces the formation of cytosolic aggregates.**

Immunofluorescence staining of mouse primary cultured neurons transfected with plasmids encoding Myc-tagged wild-type TFG, Myc-tagged p.P265A and Myc-tagged p.G269V (Green). The mcherry positive signal indicates the successfully transfected neurons. Cell nuclei were labeled with DAPI (blue). Scale bar, 10 μm.

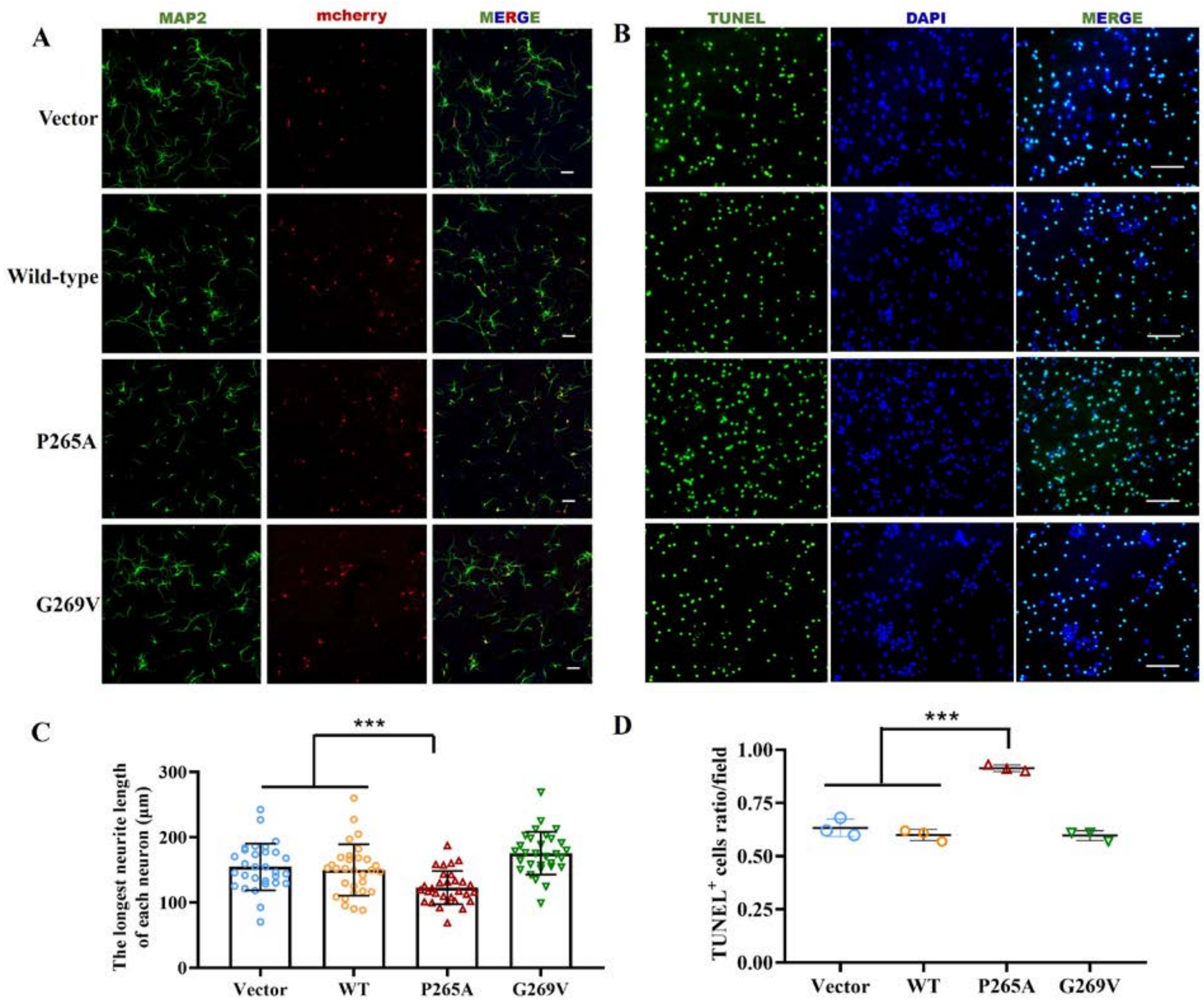


**Supplementary Figure 3. Phenotypes of TFG mutant knockin neurons.**

(A) Immunofluorescence staining of primary cultured neurons on day-17 after TFG mutant knockin. Neurons were transducted with lentivirus with mCherry (red), neurites were labeled with MAP2 (green) and the nucleus was labeled with DAPI (blue). Scale bars: 100 μm. (B) TUNEL staining of TFG mutant knockin neurons on day-17 in culture. The apoptotic cells were labeled into green and the nucleus was labeled with DAPI (blue). Scale bars: 100 μm. (C) Quantification of the longest neurite length (mean ± SD; two-tailed unpaired t-Test, ***$P < 0.001$; $n = 30$). (D) Quantification of $TUNEL^+$ cells ratio of each group (mean ± SD; two-tailed unpaired t-Test, ***$P < 0.0001$, $n = 3$).

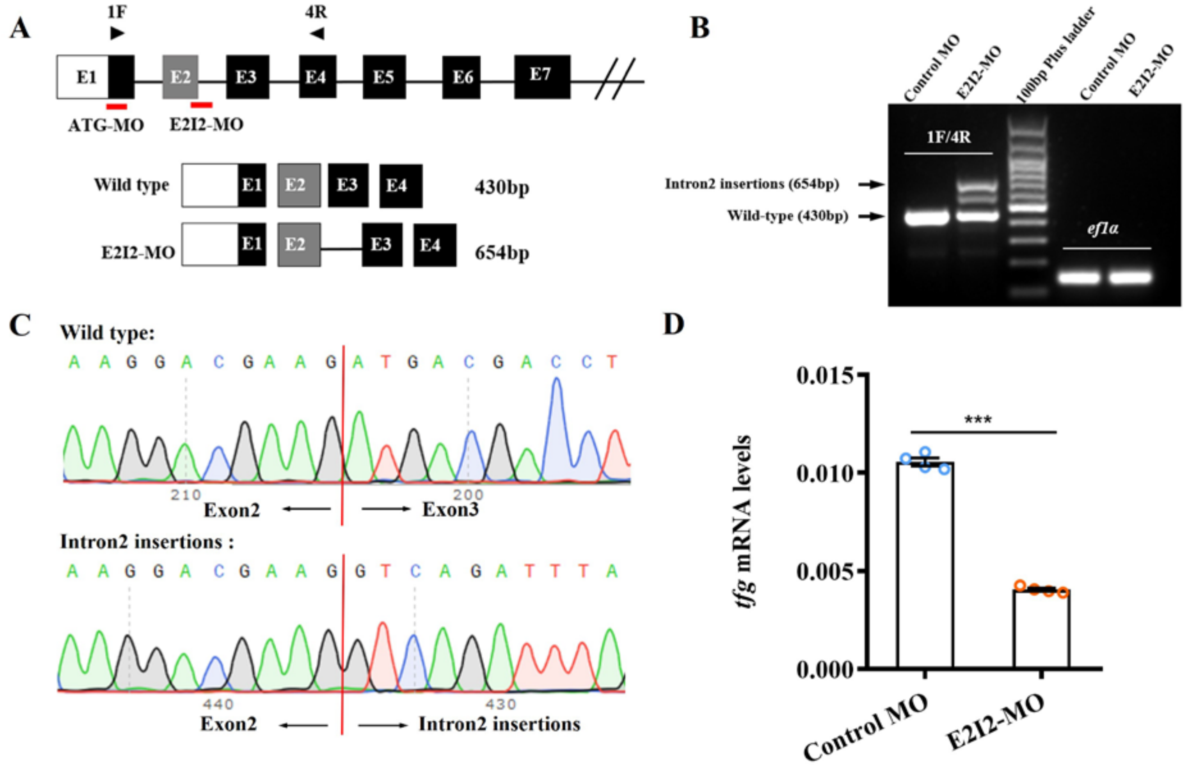


**Supplementary Figure 4. Effectiveness of *tfg* knockdown was confirmed by RT-PCR and qRT-PCR.**

(A)The zebrafish *tfg* gene was targeted by specific morpholino antisense to prevent proper splicing of exon 2 (E2I2-MO). Primers 1F and 4R interrogate the presence of wild type (non-mutant) transcripts or those in which intron 2 has been inserted. (B) RT-PCR of *tfg* transcript from control-MO and E2I2-MO morpholino-injected embryos 2 days after fertilization (dpf), demonstrating insertion of intron 2. Injection of 4ng of *tfg* morpholino alters the splicing between exon 2 and intron 2, as revealed by shift in PCR bands between control and *tfg* morpholino injected embryos. (C) Sanger sequencing of both the wild type band and the intron 2-inserted band validating the wild type sequence and the intron 2-inserted sequence. (D) Quantitative measurements of *tfg* expression levels measured by qRT-PCR at 2 dpf after introduction of 4ng of MO at the one-cell stage. (mean ± SD; two-tailed unpaired t-test, ***$P < 0.0001$, $n = 30$).

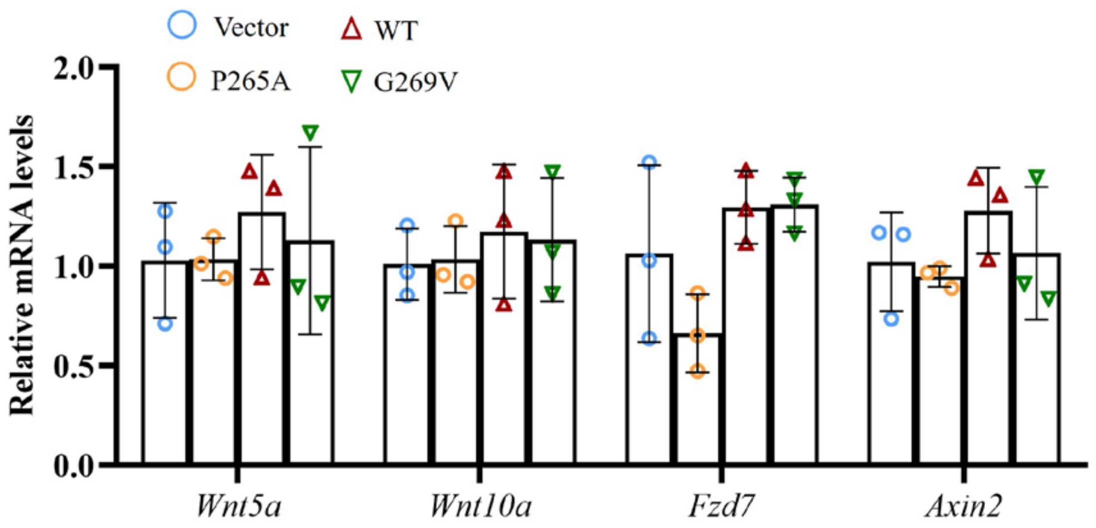


**Supplementary Figure 5. Wnt signaling is not changed in TFG mutant knockin neurons.** qRT-PCR-based validation of the key molecule in Wnt signaling in primary cultured neurons on day-12 in culture after TFG mutant knockin. (mean ± SD; two-tailed unpaired t-test; *n* = 3).

**Supplementary Method Table 1**

| **Morpholino Name** | **Morpholino oligo sequences** |
|---|---|
| *tfg*-ATG-MO (ATG-MO) | 5'-CATTCATACTGCTGGTGTTTTGGAC -3' |
| *tfg*-E3I3-MO (E3I3-MO) | 5'-TCTGAAGCCTAAATCTGACCTTCGT -3' |
| Standard Control MO | 5'- CCTCTTACCTCAGTTACAATTTATA -3' |

**Supplementary Method Table 2**

| **Gene** | **Forward Primer 5'-3'** | **Reverse Primer 5'-3'** |
|---|---|---|
| **Zebrafish** | | |
| ***tfg*** | GATATTCGCCGTATTCCTATCC | CGTCATCTTCGTCCTTGTATT |
| ***dkk1b*** | TCGCCCATGAAAACTCTACTG | TGGACCAAAAGTGACGAGC |
| ***wnt8a*** | CTCTACTCACAAAGGCTTGAGAA | CAAGACTGCAGTTTCTGGTTAAAG |
| ***wnt9a*** | CGCTACAAAATGCTGGATGG | TCAGTGGCAGAATGGACAG |
| ***lrp5*** | GCTCCTCTCTATGACCGAAAC | ACGGGCTGTTGGATGAATAA |
| ***lrp6*** | TGAGGTGCTGTTCTTCAGTAATC | GTTGGAGTCCTCGATCACAATC |
| ***frzb*** | TCCGGCTGTCTGTGTCCTCCGCTCAC | GTCCTCCTCCCGTTTGCAGCCTGGTC |
| ***fzd7a*** | CCCGCCACTATCGTGATCGCCTGCTA | CCGCGTAACAGTCCGCACGAGACATTC |
| ***fzd7b*** | CGAGCGCAAAAGGAGGCAGAT | CTGGCGCGTACATGGAGCAGA |
| ***β-catenin*** | CTGCACATTCTAGCCAGAGAC | CCTTATCCTGAGCCAGTTCAC |
| ***gsk-3β*** | ATCTTAATCCCCGCTCATGC | CAGGTTGAGGTGTTAGAGGC |
| ***axin1*** | GACATGGAGAGGAACCAGAAG | ATGACCCTGAGCTTTCTTGG |
| ***axin2*** | CTTACCCTCGGACACTTCAAG | CCCTCATACATTGGCAGAACTG |
| ***lef1*** | AATTTCTATCCCCTTTCCCCG | TGCTCCTGTTTCACCTGTG |
| ***mycn*** | CAATTGCCTCAAGTCAGTGC | ACGACATCAATCTCTTCCTCTTC |
| ***myca*** | GACACTCCACCTAACAGCTC | TCGCTTTTCCACAGTCACC |
| ***COX2*** | GTACCAACCCAAGACCTCAC | TGATGGAACAGCTCAAGAGTG |
| ***ef1α*** | GGAAATTCGAGACCAGCAAATAC | GTCGTCCAGCAGAGAATAGAAG |
| **Mouse** | | |

| | | |
|---|---|---|
| ***Ctnnb1*** | ATGGAGCCGGACAGAAAAGC | CTTGCCACTCAGGGAAGGA |
| ***Wnt5a*** | CAACTGGCAGGACTTTCTCAA | CATCTCCGATGCCGGAACT |
| ***Wnt9a*** | GGCCCAAGCACACTACAAG | AGAAGAGATGGCGTAGAGGAAA |
| ***Wnt10a*** | GCTCAACGCCAACACAGTG | CGAAAACCTCGGCTGAAGATG |
| ***Lrp5*** | AAGGGTGCTGTGTACTGGAC | AGAAGAGAACCTTACGGGACG |
| ***Lrp6*** | TTGTTGCTTTATGCAAACAGACG | GTTCGTTTAATGGCTTCTTCGC |
| ***Fzd7*** | AGACCCACCTTTCACTGCG | AAGTACATGAGGCCGTTAGCA |
| ***Mycn*** | ACCATGCCGGGGATGATCT | AGCATCTCCGTAGCCCAATTC |
| ***Lef1*** | TGTTTATCCCATCACGGGTGG | CATGGAAGTGTCGCCTGACAG |
| ***Axin1*** | CTCCAAGCAGAGGACAAAATCA | GGATGGGTTCCCCACAGAAATA |
| ***Axin2*** | TGACTCTCCTTCCAGATCCCA | TGCCCACACTAGGCTGACA |
| ***β-actin*** | GGCTGTATTCCCCTCCATCG | CCAGTTGGTAACAATGCCATGT |

**Supplementary Method Table 3**

| **ShRNA** | **Sequence** |
|---|---|
| shNC (scramble) | gCCTAAGGTTAAGTCGCCCTCGTTCAAGAGACGAGGGCGACTTAACCTTAGGc |
| sh*Tfg* | gACTGGTAGAACTTCGAAATAATTCAAGAGATTATTTCGAAGTTCTACCAGTc |
